# Scaling of species distribution explains the vast potential marine prokaryote diversity

**DOI:** 10.1038/s41598-019-54936-y

**Published:** 2019-12-10

**Authors:** Victor M. Eguíluz, Guillem Salazar, Juan Fernández-Gracia, John K. Pearman, Josep M. Gasol, Silvia G. Acinas, Shinichi Sunagawa, Xabier Irigoien, Carlos M. Duarte

**Affiliations:** 1 King Abdullah University of Science and Technology (KAUST), Red Sea Research Center (RSRC), Thuwal, 23955-6900 Saudi Arabia; 2 Instituto de Física Interdisciplinar y Sistemas Complejos IFISC (CSIC-UIB), E07122 Palma de Mallorca, Spain; 3 Department of Biology, Institute of Microbiology and Swiss Institute of Bioinformatics, ETH Zurich, Vladimir-Prelog-Weg 1-5/10, CH-8093 Zurich, Switzerland; 4 Departament de Biologia Marina i Oceanografia, Institut de Ciències del Mar-CSIC, Pg. Marítim de la Barceloneta 37-49, 08003 Barcelona, Spain; 5 AZTI - Marine Research, Herrera Kaia, Portualdea z/g, Pasaia (Gipuzkoa), 20110 Spain; 6 IKERBASQUE, Basque Foundation for Science, Bilbao, Spain

**Keywords:** Microbial ecology, Statistical physics, thermodynamics and nonlinear dynamics

## Abstract

Global ocean expeditions have provided minimum estimates of ocean’s prokaryote diversity, supported by apparent asymptotes in the number of prokaryotes with sampling effort, of about 40,000 species, representing <1% of the species cataloged in the Earth Microbiome Project, despite being the largest habitat in the biosphere. Here we demonstrate that the abundance of prokaryote OTUs follows a scaling that can be represented by a power-law distribution, and as a consequence, we demonstrate, mathematically and through simulations, that the asymptote of rarefaction curves is an apparent one, which is only reached with sample sizes approaching the entire ecosystem. We experimentally confirm these findings using exhaustive repeated sampling of a prokaryote community in the Red Sea and the exploration of global assessments of prokaryote diversity in the ocean. Our findings indicate that, far from having achieved a thorough sampling of prokaryote species abundance in the ocean, global expeditions provide just a start for this quest as the richness in the global ocean is much larger than estimated.

## Introduction

The ocean, the largest habitat in the biosphere, is a microbial-dominated ecosystem holding an estimated 10^29^ prokaryote cells^1^. Exploration of the ocean biodiversity associated with the huge prokaryote pool was prevented due to the limitations in the cultivation of marine prokaryotes^2^. This barrier was partially overcome by efficient sequencing approaches, typically targeting the genes that code for the 16S region of rDNA, which allows the definition and enumeration of the operational taxonomic units (OTUs) present in a sample, thereby providing a culture-free basis to assess biodiversity somewhat equivalent to that of species numbers^3^. In the past decade, global ocean expeditions and research based on them have utilized these technological developments in order to attempt to estimate the total number of prokaryote OTUs in the ocean^4–8^. For instance, the TARA Oceans Expedition explored prokaryote biodiversity in the upper ocean and described the detection of 35,650 prokaryote OTUs^5^ in a set of globally distributed samples, with the exception of the Arctic, while the Malaspina Expedition gave a minimum estimate of the number of prokaryote OTUs in the deep ocean which is an order of magnitude lower, at around 3,700^4^. The TARA Expedition estimated the total richness to be 37,470 OTUs based on the Chao estimator, which defines a lower bound on species richness. This result should be interpreted to be at least 37.470 OTUs in the upper ocean.

The fraction of the total volume of the ocean sampled by any study is minimal and thus requires extreme extrapolation (over 20 orders of magnitude) from the number of species found in the samples to an estimate for the global ocean. The approach used is that of rarefaction curves, a development first introduced in 1943 by Fisher *et al*. to provide a basis to estimate the species richness of Malaysian butterflies^9^, subsequently popularized by Sanders (1968)^10^ to compare benthic invertebrate species richness from marine surveys with different sample sizes. Rarefaction curves use resampling techniques to develop a curve of the number of species against the number of samples collected^11^. Initially introduced to evaluate how comprehensive the assessment of species numbers was based on a sampling set, it was subsequently used to infer the total number of species in the ecosystem investigated as that corresponding to the asymptote of the curve^12^. This approach was adopted to deliver estimates of the prokaryote species richness in the global ocean^4,5^. These estimates correspond mathematically to minimum estimates (e.g., Chao estimator)^13^, yet their precision has not been assessed. Indeed, beyond the apparent asymptote in rarefaction curves, other estimators have been proposed to estimate species richness^13–16^. Marine prokaryote communities are characterized by the presence of a few abundant OTUs and a large number of rare OTUs^2^, suggesting a much broader distribution of OTU abundance than that required to reliably apply rarefaction curves to estimate the global biodiversity of prokaryotes. Here we examine the scaling of prokaryote diversity in the ocean as a step to better understanding the extent that current assessments may underestimate prokaryote diversity in the global ocean. We do so using an array of novel approaches, including assessments across the global ocean coupled with experimental and *in silico* tests, to establish the scaling of ocean microbial diversity and explore its implications for the discovery of microbial diversity.

## Results

### Prokaryote diversity in the upper and deep ocean

**Figure 1:**
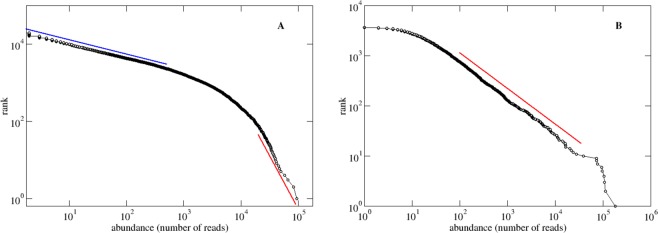
Abundance distribution of prokaryote OTUs in the upper and deep ocean. The rank *vs* abundance distribution for the (**A**) upper ocean and (**B**) deep ocean shows broad distributions with power-law tails. The abundance-rank distribution, *r* ~ *x*^−α^, where *r* is the rank of abundance *x*, has the same functional dependence (only the ranks have to be normalized between 0 and 1) as the complementary cumulative distribution CCD, CCD(*x*) = ∑_i = x,∞_ *P*(*i*), where *P*(*i*) is the abundance distribution. Thus, if the abundance rank distribution is given by *r* ~ x^−α^ the abundance distribution decays as *P*(*x*) ~ *x*^−1−α^. (**A**) For the upper ocean, the abundance distribution shows a double power-law decay separated at a characteristic scale of 2,313 reads: for abundances *x* < 2,313, the scaling exponent is 0.37 (blue line); for abundances *x* > 2,313, the scaling exponent is α = 1.57 (see Materials and Methods). (**B**) For the deep ocean, the abundance-rank distribution is characterized by a power-law decay, *P*(*x*) ~ *x*^−1−α^, with an e*x*ponent of α = 0.89 (red line).

The distribution of prokaryote OTUs in the upper ocean and deep ocean samples of the TARA Oceans^5^ and Malaspina^4^ Expeditions conform to broad distributions with power-law behavior, *P*(*x*) ~ *x*^−1−α^, where *x* represents the abundance measured in number of reads, and is characterized (the tail of the distribution) by a scaling exponent α = 1.57 for the upper ocean, and α = 0.89 for the deep ocean (Fig. 1), similar to the classic power-law describing the number of species per taxa of Willis and Yule (1922)^17^. A comparison to other broad distributions (lognormal, Weibull) shows that a distribution with a power-law tail (either pure power-law or truncated power-law) are most likely to be the best fitting (Table 1). This finding implies that the most abundant 1% OTUs account for 40% of the sequences while the least abundant 90% of sampled OTUs account for only 10% of the sequences in the upper ocean; while for the deep ocean, the most abundant 1% of OTUs account for more than 70% of the sequences while the least abundant 90% of sampled OTUs account for only 8% of the sequences.

**Table 1:** Comparing fitting models to the prokaryote abundance distribution.

	ΔAIC PL	ΔAIC TPL	ΔAIC LN	ΔAIC Weibull	α	standard error (α)	β	λ
Upper ocean	0.47	**0**	0.74	0.77	1.57	0.09	1.34	0.000019
Deep ocean	0.03	**0**	2.01	15	0.89	0.02	0.73	0.000002
Mesocosm C1	23	**0**	8.44	5.84	0.52	0.02	0.41	0.000106
Mesocosm C2	**0**	2	2.01	3.00	0.52	0.31	0.52	0
Mesocosm C3	19	**0**	13	11	0.53	0.02	0.43	0.000088
Mesocosm C4	26	**0**	8.36	5.57	0.54	0.02	0.42	0.000119
Mesocosm C5	38	**0**	13	9.49	0.57	0.02	0.38	0.000178
Mesocosm C6	18	**0**	13	11	0.52	0.02	0.44	0.000080

The delta Akaike Information Criterion (ΔAIC) indicates the most likely fit (value **0** in bold) and the difference to the most likely fit. For the six cases reported, the most likely fit is a distribution with a power-law decay (either pure or truncated). The parameters of a power law distribution *P*(*x*) ~*x* ^−1− α^ are the scaling exponent α; for the truncated power-law *P*(*x*) ~ *x* ^−1− β^ exp(−λ*x*), are the scaling exponent β, and the characteristic abundance λ (λ = 0, for a pure power-law). ΔAIC PL: delta Akaike Information Criterion for power-law distribution fit; ΔAIC TPL: delta Akaike Information Criterion for truncated power-law distribution fit; ΔAIC LN: delta Akaike Information Criterion for log-normal distribution fit; ΔAIC W: delta Akaike Information Criterion for Weibull distribution fit. The standard error of the power-law scaling exponent (α) is also reported. For the upper ocean, the prokaryote abundance distribution shows a double power-law regime. A Maximum Likelihood Estimation for a double power-law model gives *P*(*x*) ~ *x* ^−1− δ^, with exponent *δ* = 1.54 for *x* < 2,313; and P(x) ~*x* ^−1− α^, with exponent α = 0.36 for *x* ≥ 2313 (see Materials and Methods).

### Theoretical scaling

Prokaryote diversity and, in general, species diversity can be characterized by magnitudes like the Shannon and Simpson indices, which by giving greater weight to the larger, common species, provide estimators with less uncertainty^13^ (Supplementary Table 1). However, the presence of rare species impacts the estimation of species richness. Species richness scales with sampling effort as a consequence of the power-law tail of the distribution of prokaryote abundance. Let us assume that the number of OTUs of abundance *x*, *n*_*x*_, is given by *n*_*x*_ = *Ax*^−1−α^, where *A* is a normalizing constant, the scaling exponent α is larger than 0, α > 0, and the abundances are in the range *n*_*x*_ ∈ [1, *N*_max_]. Thus, the total species richness, *S*, is given by *S* = ∑_x=1,*N*max_ *n*_*x*_. In the limit of large *N*_max_, the richness can be approximated as $S=A\zeta (1+\alpha )$, that is, *A* = *S*/ζ (1 + α), where ζ (α) is the Riemann zeta function. The total number of reads *N* can be obtained by *N* = ∑_x=1,Nmax_ *x n*_*x*_. For α >1, we obtain

1 $$N=\frac{\zeta (a)}{\zeta (1+a)}$$

For α < 1, in the continuous limit $N=A{\int }_{1}^{{N}_{\max }}{x}^{-a}dx=\frac{1}{(1-a)\zeta (1+a)}S({N}_{{\max }}^{{\rm{1}}-{a}}-1)$ and the assumption that ${N}_{{\max }}^{1-\alpha }\gg 1$, we obtain

2 $$N=\frac{1}{(1-a)\zeta (1+a)}S{N}_{{\max }}^{1-a}$$

Finally, the abundance of the most abundant OTU can be evaluated as the value *N*_max_ at which there is only one group with abundance larger or equal than *N*_max_, that is, in the continuous limit ${\int }_{{N}_{{\max }}}^{\infty }{n}_{x}{dx}=1$. This leads to $S{N}_{{\max }}^{\alpha }$ (a detailed calculation can be found in ref. ^18^).

Combining the previous expressions, we obtain the following scaling laws: $S\propto {N}_{{\max }}^{\alpha }$ and for α < 1

3 $$S\propto {N}_{{\max }}^{a}\propto {{{\rm N}}}^{{\alpha }}$$

while for α > 1

4 $$S\propto {N}_{{\max }}^{a}\propto N.$$

The same scaling laws are obtained in the Yule model^19^ (which can also be mapped to the Simon model^20,21^), where the scaling exponent α is related to the ratio between speciation rate *g* and group growth *s*, $\alpha$ = *g*/*s*. Systems showing distributions with power-law tails are ubiquitous: several methodologies have been described to fit and compare different functional forms as well as mechanisms to explain their origin^18,22–24^.

### Empirical and *in silico* scaling

**Figure 2:**
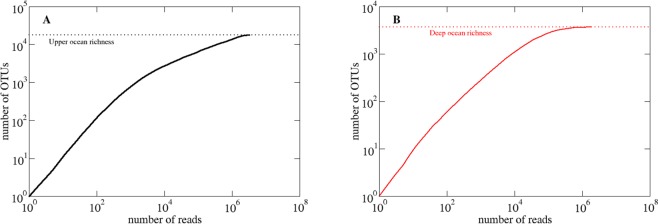
Number of species as a function of the number of reads. The expected number of OTUs in a random sampling of the total population grows sublinearly with sampling size, *S* ~ *N*^γ^. (**A**) In the upper ocean (continuous black line), we can identify a first quasi-linear regime with γ = 0.90 (confidence interval 95% <0.01) and a second regimen with γ = 0.33 (confidence interval <0.01), while (**B**) in the deep ocean (continuous red line) the exponent γ = 0.62 (confidence interval <0.01). The number of OTUs in the upper ocean (horizontal dotted black line) is estimated at 35,650 OTUs^5^ and in the deep ocean (horizontal dotted red line) the maximum number of OTUs found is 3,695^4^.

The scaling of species richness and the distribution of species abundances are two sides of the same coin. The power-law distribution of prokaryote species abundance implies that species richness (*S*) scales with sampling effort (*N*, number of samples) as *S* ~ *N*^γ^, where (i) γ equals the exponent of the rank-abundance power-law (i.e., γ = α), when this exponent is α < 1, as observed in the deep ocean (Malaspina Oceans Expedition, Fig. 2), and (ii) *S* is proportional to sampling effort (i.e., γ = 1) for larger exponents α > 1, such as observed for the upper ocean (TARA Expedition, Fig. 2). Indeed, the power-law scaling of species richness with sampling effort implicit in the power-law distribution of the prokaryote species abundance distribution (Fig. 1) implies that the asymptote of rarefaction curves is artifactual and that indeed, the number of species does not approach any asymptote at the sampling effort this far deployed by global expeditions (Fig. 2). This expectation was confirmed by producing an *in silico* global ocean microbiome with an underlying distribution of prokaryote species abundance with the same shape and exponent as those empirically derived for the upper and deep ocean (dotted lines in Fig. 2). The *in silico* data was obtained, first, by expanding the empirically fitted data to larger populations and, second, by randomly generating abundance OTUs from the expanded distributions (see Materials and Methods). These simulations showed that increasing sampling effort, expressed as the total number of 16S reads sequenced, about 30 to 50 times relative to that applied to the upper and deep ocean by the TARA Oceans (3.3 × 10^6^ reads, ref. ^5^) and Malaspina Expedition (1.8 × 10^6^ reads, ref. ^4^) respectively would lead to estimates of prokaryote species abundance 4.2 and 1.2 times greater than inferred on the basis of rarefaction curves for the upper and deep ocean respectively (Fig. 2 and Supplementary Fig. 1). The estimators are calculated for a global population of 10^8^ reads, which corresponds to 1 liter of upper ocean water (10^5^ prokaryote cells/ml) and 10 liters of deep ocean water (10^4^ prokaryote cells/ml) (Supplementary Table 1).

### Mesocosm experiment

**Figure 3:**
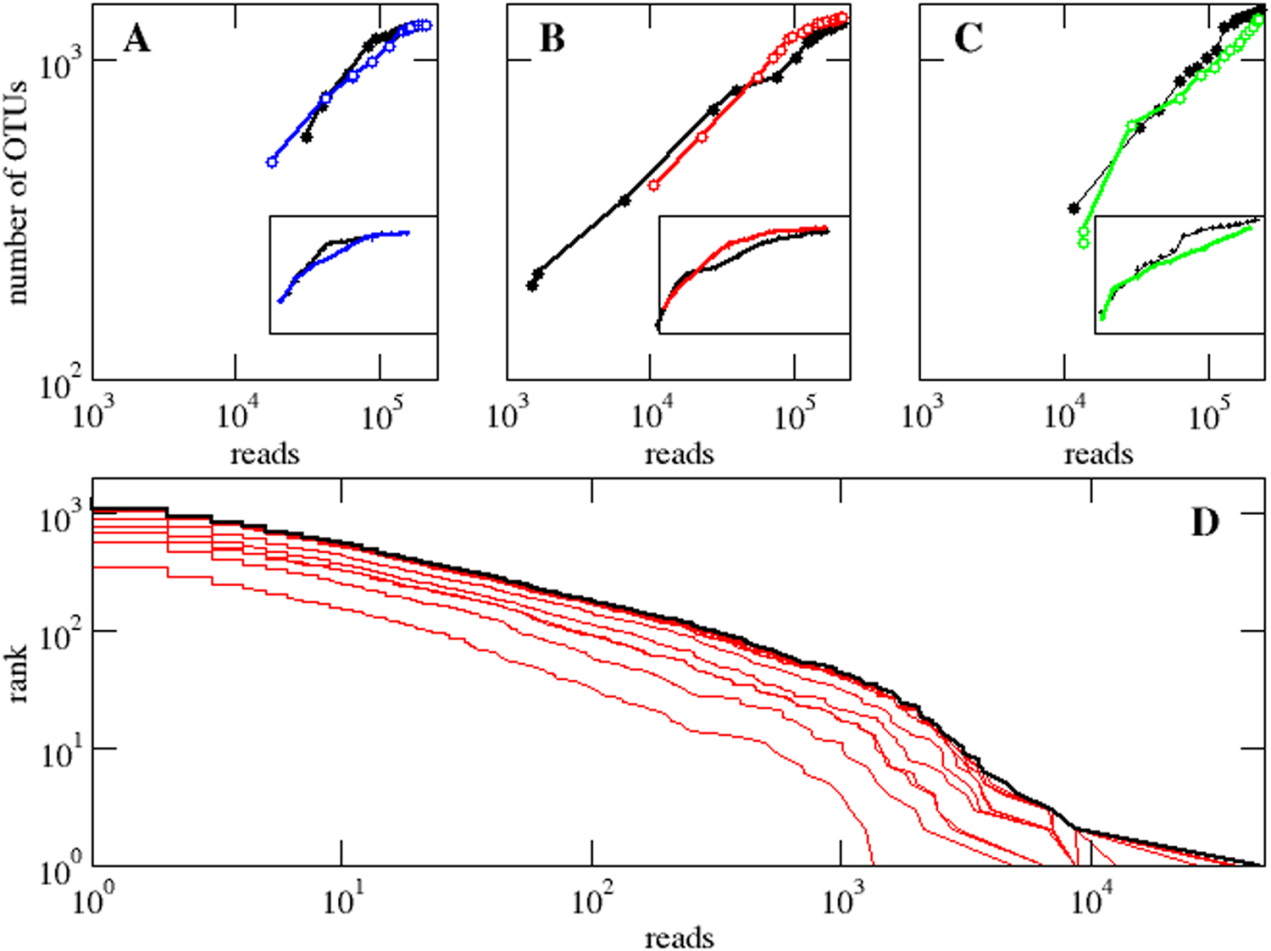
Scaling of the number of OTUs with the number of reads in an experiment. The number of prokaryote OTUs as a function of the number of reads is plotted, in a log-log scale, every two days as the experiment runs for 20 days in different conditions (**A**) control (Mesocosm C1 and C2), (**B**) single dose nitrate phosphate addition (NP) (Mesocosm C3 and C4), and (**C**) single dose nitrate phosphate sulfate addition (NPS) (Mesocosm C5 and C6). For all the conditions, we plot two replicates. The number of OTUs, *S*, scales with the number of reads, *N*, as *S* ~ *N*^γ^, with γ = 0.44, 0.40 (control), 0.38, 0.40 (NP), 0.48, 0.52 (NPS). The insets show the same data in linear scale (same ranges as main plots) where an apparent saturation asymptote is observed. (**D**) Abundance vs rank plot for one of the controls for successive days from bottom to top. The exponent of a power-law distribution fit, *P*(*x*) ~ *x*^−1−α^, for the aggregated data after 20 days (black line) is α = 0.52.

We challenged the mathematically-derived predictions, tested and confirmed by the *in silico* experiment, by enclosing a plankton community of the Central Red Sea in duplicate, and sampling and sequencing it every day during 20 days^25^ (c.f. Materials and Methods). The abundance distribution of prokaryote OTUs in the sampled Central Red Sea community continued to increase with additional sampling effort (Fig. 3), according to a power-law distribution with an average exponent of α = 0.53, comparable to that obtained for the deep ocean (α = 0.89) and for the less abundant of the upper ocean (α = 0.36) (Fig. 3D). In line with the upper and deep ocean cases, a comparative analysis performed for all the samples of the mesocosm experiment in three experimental conditions (control, single dose Nitrate-Phosphate addition and single dose Nitrate-Phosphate-Silicate addition) shows that a distribution with a power-law decay (either as a pure power-law or a truncated power-law) is the most likely fit (Supplementary Tables 2–7). The results confirmed the expectation that the number of OTUs retrieved in this community increased, on average, with the power 0.46 of the cumulative number of 16S reads sequenced without a clear asymptotic behavior despite exhaustive sampling (Fig. 3A–C and Tables 1 and 2).

**Table 2:** Scaling exponents and confidence interval for the mesocosm experiment.

	Scaling exponent γ	Confidence Interval (95%)	Days of observation
Mesocosm C1	0.44	0.026	14
Mesocosm C2	0.70	0.089	18
Mesocosm C3	0.44	0.039	19
Mesocosm C4	0.29	0.043	17
Mesocosm C5	0.36	0.062	19
Mesocosm C6	0.54	0.076	20

For each condition and for each replica of the mesocosm experiment, the number of prokaryote species is fitted with the number of reads *S* ~ *N*^γ^, with a least square method and the confidence intervals are calculated according to the number of days of observations in each condition.

## Discussion

The results presented show that the abundance of different prokaryotic species in the ocean is described by a power-law distribution that implies that the total number of OTUs continues to increase, with a power given by that of the rank-abundance power-law, with increasing sampling effort. The dependence of the estimated richness on sampling effort is not an exclusive property of a power-law distribution and it has also been reported for lognormal distributions both theoretically^26^ and empirically^7,23^. We expect that the effort-dependence of the species richness applies to distributions with sufficient long tails and thus characterized by the presence of many rare species (OTUs). Thus, in the presence of a rare biosphere^2^, the effort-dependence of richness estimates is the expected outcome. Hence, the estimates that the upper and deep ocean contain ca. 37,000 and 3,700 prokaryote OTUs^4,5^, respectively, derived from rarefaction curves is an underestimate (Fig. 2). The estimation of the diversity based on sampling effort (both the number of samples collected and the sequencing depth applied to each sample) still represents a challenge and requires broad extrapolations. We have addressed the estimation of prokaryote diversity with the parsimonious assumption that the sampled distribution represents the population distribution, furthermore supported by the relatively conserved shape of this abundance distributions when sampling is replicated as in our mesocosm experiment (see Supplementary Tables 2–7). Thus, we have explored the estimation of prokaryote diversity derived from fitting different underlying distributions to the upper and deep ocean, and the mesocosm experiment. Future research increasing sampling effort, both for individual communities and locations across the ocean, are likely to yield OTU counts much higher than these estimates. The power-law distribution of species richness is not a new observation in ecology^27–31^ but is rooted in the seminal work of Willis and Yules showing a power-law distribution of species membership within taxa^17^. Indeed, a recent estimate of oceanic prokaryote species richness derived by extrapolating across more than 20 orders of magnitude the relationship between species numbers and number of cells sampled to match the 10^29^ prokaryote cells estimated in the global ocean, led to an estimate of 10^10^ different OTUs for this ecosystem^7^. Whereas the estimate derived from such wild extrapolation rests on a number of assumptions and does not necessarily reflect the shape of species abundance distribution of oceanic prokaryotes, it supports our empirical, mathematical, modeling and experimental results that indicate that the number of prokaryote OTUs in the ocean is far larger than currently estimated. A much-enhanced sampling effort is, therefore, required to unveil the prokaryote diversity concealed within the rare biosphere. Enhanced sampling efforts should be deployed both to retrieve the least abundant components of anyone community and also to benefit from the dynamics of microbial populations, which can bring otherwise rare components of the microbial biosphere to a level of abundance where they may be retrieved in sequencing projects (e.g., ref. ^32^). Efforts to achieve an inventory of prokaryotic OTUs in the ocean will require a far more exhaustive sampling than deployed to date combined with sound extrapolation approaches rooted in the observed abundance distributions of prokaryotic OTUs.

## Materials and Methods

### Data and experimental design

We have analyzed three datasets. The three empirical datasets are: from the TARA expedition we collected the abundance of 18,022 OTUs from the surface water and deep chlorophyll maximum layers in 63 and 46 sites, respectively, containing 3,323,839 reads^5^ (available at http://ocean-microbiome.embl.de/companion.html). From the Malaspina expedition, we collected the abundance of 3,695 free-living and particle-attached OTUs from 30 globally distributed sites in the bathypelagic ocean^4^ (available at https://github.com/GuillemSalazar/MolEcol_2015). The experimental data reported the OTU abundance every day for a period of 20 days in three experimental conditions: (a) control (referred as Mesocosm C1 and C2), (b) single dose Nitrate-Phosphate addition (referred as C3 and C4), and (c) single dose Nitrate-Phosphate-Silicate addition (referred as C5 and C6) (Nitrate = 2 µM, Phosphate = 0.12 µM, Silicate = 3.75 µM)^25^. Samples range from an average of 11,126 ± 5,400 (SD) reads leading to 337 ± 100 (SD) OTUs the first day to an aggregated number of 212,761 ± 22,000 (SD) reads and 1,331 ± 56 (SD) OTUs after completion of the experiment. Raw reads, which the OTUs counts were based on, have been deposited in the NCBI Sequence Read Archive under the accession number SRP051855.

### Statistical analysis

#### Abundance distribution

The model fittings of the power-law distributions, the truncated power-law distributions, lognormal distributions, and the stretched exponential distributions ware obtained with the Maximum Likelihood Estimation applied to the empirical data^33^. For the upper ocean, we have fitted also a double power-law distribution.

#### *In silico* prokaryote diversity: upper ocean

We proposed a distribution with two power-law regimes, with the parameter values (scaling exponents and transition point) obtained as described below: *P*(*x*) = *Ax*^−1−*δ*^, for abundances x ≤ x_c_, and *P*(*x*) = *Bx*^−1−α^, for *x* > *x*_c_. The condition that the distribution is continuous at x_c_ (P(x_c_) = *Ax*_*c*_^−1−*δ*^ = *Bx*_*c*_^−1−α^) and the normalization (Σ*P*(*x*) = 1), lead to the values *A* = *δ* + (*δ* – α) *x*_c_^−α^, and *B* = A*x*_c_^(*δ* –α)^. We assigned to the exponents α and δ, and to the transition point *x*_c_ the values obtained from the Maximum Likelihood α = 1.54, δ = 0.36, and *x*_c_ = 2,313.

#### *In silico* prokaryote diversity: deep ocean

We proposed a shifted power-law to capture the power-law tail and the deviation at the head of the distribution: P(x) = α ((x + x_0_)/(1 + x_0_))^−1− α^. The parameters α and *x*_0_ can be obtained by the Maximum Likelihood Estimation: α = N_OTU_ Σlog ((*x*_0_ + *x*_*i*_)/(1 + *x*_0_)), and (*x*_0_ + 1) Σ1/(1 + *x*_*i*_) = N_OTU_ α /(1 − α). To solve these implicit equations, we proposed *x*_0_ and α, evaluate the previous expressions, and obtained new values *x*_0_′ and α′. We repeated these steps until we reached the condition |*x*_0_′ − *x*_0_| < *T*, for some convergence value *T*. For *T* = 10^−6^, the values we obtained are α = 0.89, and *x*_0_ = 20.34.

#### Akaike Information Criterion (AIC)

The Akaike Information Criterion is defined as AIC = −2log *L* + 2 *V*, where *L* is the maximum likelihood of a fit model, and *V* is the number of free parameters. The delta Akaike Information Criterion is calculated as ΔAIC = AIC-AIC_min_, where AIC_min_ corresponds to the minimum value of all the candidate models, and AIC the value of the candidate model. The weight AIC

$${w}_{i}(AIC)=\frac{{\exp }(\frac{-1}{2}{\Delta }_{{i}}{\rm{AIC}})}{{\sum }_{K=1}^{M}{\exp }(\frac{-1}{2}{\Delta }_{{k}}{\rm{AIC}})}$$

can be interpreted as the probability that the model is the best model (in the AIC sense, that it minimizes the Kullback–Leibler discrepancy), given the data and the set

of candidate models (e.g., Burnham & Anderson, 2001).

#### Extrapolation of abundance distributions for larger number of samples

For the upper Ocean, the abundance distribution is fitted to a double power-law defined as *P*(*x*) = *Ax*^*−*1−*δ*^ for *x* < *x*_*c*_ and *P*(*x*) = *Bx*^−1−*α*^ for *x*_*c*_ < *x*. A continuity condition (*Ax*_*c*_^−1−*δ*^ = *Bx*_*c*_^−1−*α*^) and the normalization condition (1 = ∫_1_^*∞*^*P*(*x*)*dx*) gives the values for the constants *A* and *B* as *A* = *αδ*(*α* + (*δ* *−* *α*)*x*_*c*_^*−δ*^)^−1^ and *B* = *A x*_*c*_^*α*−*δ*^. In order to fit this distribution, we have to obtain estimates for the two exponents *δ* and *α* and for the cutoff *x*_*c*_. We use first the maximum likelihood method implemented in ref. ^30^ which fits the exponent for the tail *α* and the value of the cutoff *x*_*c*_. Then we adjust the value of the exponent for the range [1, *x*_*c*_] by using the same method, only fixing the minimum value to 1 and disregarding any data over the cutoff value *x*_*c*_. In order to extract the behavior of the parameters for an increasingly large ecosystem, we used increasingly randomly aggregated samples from the TARA Oceans Expedition (139 samples in total). The average parameters for aggregations of samples of similar total number of reads are shown in the left column of Supplementary Fig. 2 in black and the error bars reflect their standard deviation. Next, in order to extrapolate these parameters to larger number of reads we fitted the estimated parameters to some simple curves (shown in red in Supplementary Fig. 2). The results were *x*_*c*_** = **0.0002 · *N*_*reads*_^1.1^ + 52.6, *δ* = 0*.32* (1 + 0.71 exp(*−N*_*reads*_*/570007*)) and *α* = 1.42 (1 − 0.2 exp(*−N*_*reads*_*/110185*)). Note that the values of the scaling exponent of the tail of the distribution *α* are in agreement with recently reported estimates^34^. For the *in-vitro* generation of larger samples we extrapolated the parameter values to the value corresponding to the desired number of reads and generated random numbers from the corresponding distribution up to the desired number of reads, using the method of the inversion of the cumulative distribution.

For the deep Ocean, the abundance distribution is fitted to a shifted power-law *P*(*x*) = A(*x* + *x*_0_)^−*1*−*α*^ with a maximum possible value for the abundance *x*_max_. The value of A is given by the normalization condition (1 = ∫_1_^*Xmax*^*P*(*x*)*dx*) and is A = *α*((1 + *x*_0_)^−*α*^ − (*x*_max_ + *x*_0_)^−*α*^)^−1^. In this case, we need to estimate again three parameters to fit the distribution. In order to estimate the parameters, we first fitted the exponent *α* and the shifting parameter *x*_0_ by solving iteratively the equations from maximum likelihood:

$$a=S{(\mathop{\sum }\limits_{i=1}^{S}\log \frac{({{x}}_{{i}}+{{x}}_{{\rm{0}}})}{1+{{x}}_{{\rm{0}}}})}^{-1}$$

$${x}_{0}=aS{((1+a){\sum }_{i=1}^{s}\frac{1}{{x}_{i}+{x}_{0}})}^{-1},$$

where *S* stands for the number of data points. With those estimated parameters we estimated the maximum abundance *x*_max_ through the average abundance <*x*> found in the data by solving the implicit equation <*x*> = ∫_1_^*Xmax*^*xP*(*x*)*dx:*

$$\langle x\rangle =\frac{a}{1-a}\frac{{({x}_{{\max }}+{x}_{0})}^{1-a}-{(1+{x}_{0})}^{1-a}}{{(1+{x}_{0})}^{-a}-{({x}_{{\max }}+{x}_{0})}^{-a}}-{x}_{0}$$

The parameters are shown in the right column of Supplementary Fig. 2 and again in black are average estimates with standard deviations shown with error bars, and in red the simple fitted curves used for the extrapolation. In this case the simple curves fitted were *x*_*0*_ = 0.000003 *N*_*reads*_^1.1^ – 1, *α* = 0.88 (1 − 0.45 exp(*−N*_*reads*_/363263)) and <*x*> = 0.00042 *N*_*reads*_^0.97^ + 23.6.

The estimation for a larger number of reads was performed as for the upper ocean but using the proper shifted power-law distribution as given by the extrapolated parameters.

## Supplementary information

Supplementary Figures

## Data Availability

The TARA expedition dataset is available at http://ocean-microbiome.embl.de/companion.html; the Malaspina expedition dataset is available at https://github.com/GuillemSalazar/MolEcol_2015; and the experimental data have been deposited in the NCBI Sequence Read Archive under the accession number SRP051855.
