# Supplementary material for: Scaling of species distribution explains the vast potential marine prokaryote diversity
Source: Sci Rep. 2019 Dec 10;9:18710. doi: 10.1038/s41598-019-54936-y (PMC6904450; doi:10.1038/s41598-019-54936-y)
Supplement: Supplementary file 1 — Supplementary Figures [file 41598_2019_54936_MOESM1_ESM.pdf]

Supplementary Information for

Scaling of species distribution explains the vast potential marine prokaryote diversity

Victor M. Eguíluz, Guillem Salazar, Juan Fernández-Gracia, John K. Pearman, Josep M. Gasol, Silvia G. Acinas, Shinichi Sunagawa, Xabier Irigoien and Carlos M. Duarte

This PDF file includes:

- Supplementary Table 1. Characterization of the prokaryote diversity and estimators
- Supplementary Table 2: Comparing fitting models to the prokaryote abundance distribution for the mesocosm experiment in the control condition (C1).
- Supplementary Table 3: Comparing fitting models to the prokaryote abundance distribution for the mesocosm experiment in the control condition (C2).
- Supplementary Table 4: Comparing fitting models to the prokaryote abundance distribution for the mesocosm experiment in the single dose Nitrate-Phosphate addition condition (C3).
- Supplementary Table 5: Comparing fitting models to the prokaryote abundance distribution for the mesocosm experiment in the single dose Nitrate-Phosphate addition condition (C4).
- Supplementary Table 6: Comparing fitting models to the prokaryote abundance distribution for the mesocosm experiment in the single dose Nitrate-Phosphate-Silicate addition condition (C5).
- Supplementary Table 7: Comparing fitting models to the prokaryote abundance distribution for the mesocosm experiment in the single dose Nitrate-Phosphate-Silicate addition condition (C6).
- Supplementary Fig. 1. Estimation of the prokaryote diversity for the (A) upper and (B) deep ocean.
- Supplementary Fig. 2**.** Fitting of parameters for the distribution of prokaryote diversity.

	Number of reads	Species Richness	D_1	D_2	D_-	D_+	*E*
Upper Ocean	3,323,839	18,022	1,486.4	477.4	24,442	172,451	76,579
Deep Ocean	1,789,427	3,695	100.3	29.1	3,749	8,445	4,435

Supplementary Table 1. Characterization of prokaryote diversity and estimators. The diversity of the prokaryote populations in the marine samples are reported as number of reads, species richness, and Hill diversity $\boldsymbol{D_1 = exp(\sum p_i log p_i)}$, and $\boldsymbol{D_2 = \left(\sum p_i^2\right)^{-1}}$. Shannon diversity, Sh, is related to Hill diversity D_1, $\boldsymbol{D_1 = exp(\mathrm{Sh})}$; Simpson diversity, Si, is related to Hill diversity D_2, $\boldsymbol{D_2 = (\mathrm{Si})^{-1}}$. Hill diversities provide an estimation of the effective population. The estimation of the prokaryote diversity is calculated for a total population of 10^8 reads which correspond to 1 liter of upper ocean (10^5 prokaryote cells/ml) and 10 liters of deep ocean water (10^4 prokaryote cells/ml). The minimum D_- and maximum D_+ estimators are calculated applying the MATLAB code provided by Ref. (13) to the empirical data. The estimator *E* is based on the average values obtained by expanding the empirically fitted model distributions (see Material and Methods; see also Supplementary Figure 1).

	AIC PL	AIC TPL	AIC LN	AIC W	α	standard error (α)	β	λ
Day 3	**0**	1.76	2.01	3.71	0.83	0.08	0.81	0.000022
Day 5	15	**0**	25	26	0.49	0.03	0.46	0.001203
Day 6	11	**0**	18	18	0.54	0.04	0.52	0.00182
Day 7	15	**0**	33	30	0.44	0.02	0.40	0.000667
Day 9	**0**	1.44	1.79	1.75	1.34	0.25	1.05	0.000377
Day 11	4.13	**0**	14	12	0.60	0.03	0.46	0.002256
Day 12	**0**	0.63	0.88	0.97	2.17	0.69	0.72	0.571927
Day 13	11	**0**	14	18	0.51	0.04	0.56	0.000029
Day 14	**0**	1.13	4.60	6.96	0.62	0.05	0.62	0.000033
Day 15	**0**	1.13	4.73	4.65	0.69	0.06	0.69	0.000062
Day 16	**0**	1.25	5.60	5.88	0.65	0.05	0.62	0.000323
Day 17	10	**0**	21	19	0.49	0.03	0.49	0.000425
Day 18	**0**	0.93	1.91	1.75	0.72	0.09	0.59	0.000964
Day 20	**0**	0.62	6.13	7.05	0.55	0.04	0.51	0.000147

Supplementary Table 2: Comparing fitting models to the prokaryote abundance distribution for the mesocosm experiment in the control condition (C1). The delta Akaike Information Criterion is reported for the prokaryote diversity obtained for the indicated day. The delta Akaike Information Criterion (ΔAIC) indicates the most likely fit (value **0** in bold) and the difference to the most likely fit. For the six cases reported, the most likely fit is a distribution with a power law decay (either pure or truncated). The parameters of a power law distribution $P(x) \sim x^{-1-\alpha}$ are the scaling exponent α; for the truncated power law $P(x) \sim x^{-1-\beta} \exp(-\lambda x)$, are the scaling exponent β, and the characteristic abundance λ (λ=0, for a pure power law). ΔAIC PL: delta Akaike Information Criterion for power-law fit; ΔAIC TPL: delta Akaike Information Criterion for truncated power-law fit; ΔAIC LN: delta Akaike Information Criterion for log-normal fit; ΔAIC W: delta Akaike Information Criterion for Weibull fit. The standard error of the power law scaling exponent (α) is also reported.

	AIC PL	AIC TPL	AIC LN	AIC W	α	standard error (α)	β	λ
Day 1	0.68	**0**	1.99	1.85	0.85	0.09	0.68	0.000442
Day 2	1.08	**0**	2.99	3.09	0.67	0.04	0.59	0.000269
Day 3	10	**0**	9.74	8.28	0.59	0.03	0.43	0.000889
Day 4	6.14	**0**	13	12	0.56	0.04	0.43	0.009292
Day 6	14	**0**	31	28	0.42	0.02	0.38	0.000485
Day 7	8.86	**0**	15	13	0.49	0.02	0.37	0.000569
Day 8	8.36	**0**	19	16	0.53	0.02	0.42	0.000857
Day 9	9.36	**0**	19	18	0.51	0.03	0.43	0.003876
Day 11	**0**	2.18	2.61	2.57	1.28	0.34	0.37	0.161714
Day 12	7.3	**0**	15	14	0.48	0.03	0.48	0.000366
Day 13	**0**	1.58	2.32	3.79	0.85	0.11	0.83	0.001454
Day 14	8.45	**0**	13	12	0.56	0.05	0.60	0.000354
Day 16	**0**	0.92	2.37	6.18	0.72	0.07	0.73	0.000043
Day 17	**0**	1.35	4.43	5.71	0.58	0.05	0.58	0.000021
Day 18	**0**	1.43	4.05	5.02	0.60	0.06	0.60	0.000029
Day 19	**0**	0.69	1.81	8.15	1.00	0.15	1.05	0
Day 20	**0**	1.02	1.5	6.62	0.77	0.09	0.79	0

Supplementary Table 3: Comparing fitting models to the prokaryote abundance distribution for the mesocosm experiment in the control condition (C2). The delta Akaike Information Criterion is reported for the prokaryote diversity obtained for the indicated day. The delta Akaike Information Criterion (ΔAIC) indicates the most likely fit (value **0** in bold) and the difference to the most likely fit. For the six cases reported, the most likely fit is a distribution with a power law decay (either pure or truncated). The parameters of a power law distribution $P(x) \sim x^{-1-\alpha}$ are the scaling exponent α; for the truncated power law $P(x) \sim x^{-1-\beta} \exp(-\lambda x)$, are the scaling exponent β, and the characteristic abundance λ (λ=0, for a pure power law). ΔAIC PL: delta Akaike Information Criterion for power-law fit; ΔAIC TPL: delta Akaike Information Criterion for truncated power-law fit; ΔAIC LN: delta Akaike Information Criterion for log-normal fit; ΔAIC W: delta Akaike Information Criterion for Weibull fit. The standard error of the power law scaling exponent (α) is also reported.

	AIC PL	AIC TPL	AIC LN	AIC W	α	standard error (α)	β	λ
Day 1	**0**	2.05	3.43	3.96	0.91	0.1	0.86	0.001474
Day 2	**0**	1.6	2.4	2.25	1.28	0.31	1.18	0.022295
Day 3	13	**0**	24	22	0.53	0.03	0.52	0.001254
Day 4	0.31	**0**	10	8.81	0.58	0.03	0.53	0.000312
Day 6	18	**0**	35	34	0.48	0.02	0.44	0.001086
Day 7	16	**0**	22	24	0.50	0.03	0.54	0.000015
Day 8	16	**0**	35	32	0.42	0.02	0.37	0.000469
Day 9	19	**0**	40	36	0.45	0.02	0.41	0.000703
Day 11	16	**0**	31	31	0.46	0.02	0.41	0.001102
Day 12	11	**0**	23	21	0.46	0.03	0.34	0.003312
Day 13	3.25	**0**	11	10	0.54	0.03	0.43	0.000794
Day 14	14	**0**	29	28	0.43	0.02	0.39	0.000457
Day 15	3.41	**0**	7.59	6.45	0.53	0.05	0.29	0.016315
Day 16	2.7	**0**	8.45	7.89	0.56	0.04	0.42	0.001829
Day 17	12	**0**	25	24	0.46	0.02	0.43	0.000744
Day 18	**0**	0.51	6.79	6.5	0.59	0.04	0.53	0.000403
Day 19	**0**	1.85	2.1	3.78	0.76	0.16	0.74	0.00052
Day 20	9.58	**0**	22	20	0.46	0.03	0.41	0.001219

Supplementary Table 4: Comparing fitting models to the prokaryote abundance distribution for the mesocosm experiment in the single dose Nitrate-Phosphate addition condition (C3). The delta Akaike Information Criterion is reported for the prokaryote diversity obtained for the indicated day. The delta Akaike Information Criterion (ΔAIC) indicates the most likely fit (value **0** in bold) and the difference to the most likely fit. For the six cases reported, the most likely fit is a distribution with a power law decay (either pure or truncated). The parameters of a power law distribution $P(x) \sim x^{-1-\alpha}$ are the scaling exponent α; for the truncated power law $P(x) \sim x^{-1-\beta} \exp(-\lambda x)$, are the scaling exponent β, and the characteristic abundance λ (λ=0, for a pure power law). ΔAIC PL: delta Akaike Information Criterion for power-law fit; ΔAIC TPL: delta Akaike Information Criterion for truncated power-law fit; ΔAIC LN: delta Akaike Information Criterion for log-normal fit; ΔAIC W: delta Akaike Information Criterion for Weibull fit. The standard error of the power law scaling exponent (α) is also reported.

	AIC PL	AIC TPL	AIC LN	AIC W	α	standard error (α)	β	λ
Day 1	**0**	0.68	1.79	1.88	0.89	0.09	0.78	0.000478
Day 2	2.19	**0**	2.73	2.38	0.71	0.06	0.54	0.000694
Day 3	8.7	**0**	4.43	3.33	0.66	0.04	0.49	0.000528
Day 4	2.24	**0**	3.39	2.87	0.65	0.04	0.54	0.000529
Day 5	20	**0**	36	35	0.51	0.02	0.46	0.002391
Day 6	0.62	**0**	1.66	1.46	0.94	0.08	0.76	0.001047
Day 7	6.65	**0**	16	14	0.46	0.03	0.41	0.001157
Day 8	15	**0**	27	26	0.48	0.03	0.48	0.000352
Day 9	12	**0**	26	24	0.46	0.02	0.42	0.000644
Day 10	**0**	0.46	6.91	9.91	0.58	0.03	0.54	0.000192
Day 11	**0**	1.85	1.94	2.01	1.70	0.76	0.79	0.251862
Day 12	4.36	**0**	11	9.75	0.47	0.04	0.49	0.000175
Day 13	**0**	1.23	2.73	2.93	0.53	0.06	0.49	0.000067
Day 14	9.6	**0**	19	17	0.48	0.03	0.49	0.000131
Day 16	6.58	**0**	14	12	0.54	0.04	0.47	0.004884
Day 17	**0**	1.37	2.89	4.37	0.69	0.06	0.63	0.000272
Day 18	8.78	**0**	20	18	0.45	0.03	0.40	0.000718
Day 19	**0**	1.11	3.31	2.95	0.63	0.05	0.57	0.000346
Day 20	4.42	**0**	11	9.43	0.50	0.04	0.49	0.000815

Supplementary Table 5. Comparing fitting models to the prokaryote abundance distribution for the mesocosm experiment in the single dose Nitrate-Phosphate addition condition (C4). The delta Akaike Information Criterion is reported for the prokaryote diversity obtained for the indicated day. The delta Akaike Information Criterion (ΔAIC) indicates the most likely fit (value **0** in bold) and the difference to the most likely fit. For the six cases reported, the most likely fit is a distribution with a power law decay (either pure or truncated). The parameters of a power law distribution $P(x) \sim x^{-1-\alpha}$ are the scaling exponent α; for the truncated power law $P(x) \sim x^{-1-\beta} \exp(-\lambda x)$, are the scaling exponent β, and the characteristic abundance λ (λ=0, for a pure power law). ΔAIC PL: delta Akaike Information Criterion for power-law fit; ΔAIC TPL: delta Akaike Information Criterion for truncated power-law fit; ΔAIC LN: delta Akaike Information Criterion for log-normal fit; ΔAIC W: delta Akaike Information Criterion for Weibull fit. The standard error of the power law scaling exponent (α) is also reported.

	AIC PL	AIC TPL	AIC LN	AIC W	α	standard error (α)	β	λ
Day 1	14	**0**	24	24	0.48	0.03	0.47	0.000496
Day 2	19	**0**	37	33	0.42	0.02	0.34	0.000939
Day 3	18	**0**	29	24	0.43	0.02	0.30	0.002022
Day 4	0.57	**0**	9.58	12	0.58	0.03	0.52	0.000409
Day 5	17	**0**	34	32	0.46	0.02	0.38	0.001897
Day 6	11	**0**	23	21	0.47	0.03	0.43	0.00085
Day 7	9.37	**0**	21	17	0.43	0.02	0.38	0.000595
Day 9	15	**0**	30	27	0.42	0.02	0.33	0.001075
Day 10	4.22	**0**	3.14	2.46	0.79	0.05	0.61	0.001151
Day 11	6.59	**0**	13	12	0.50	0.03	0.38	0.000727
Day 12	0.7	**0**	5.75	5.33	0.69	0.05	0.56	0.001667
Day 13	0.26	**0**	4.86	4.16	0.63	0.04	0.55	0.000472
Day 14	4.94	**0**	7.21	6.42	0.66	0.04	0.50	0.001278
Day 15	**0**	0.61	4.91	5.79	0.67	0.04	0.60	0.000641
Day 16	17	**0**	30	29	0.53	0.03	0.45	0.004826
Day 17	**0**	0.24	1.34	1.17	1.11	0.14	0.80	0.001952
Day 18	**0**	1.3	3.25	4.56	0.87	0.07	0.77	0.001157
Day 19	0.25	**0**	2.12	1.95	0.91	0.06	0.78	0.001034
Day 20	2.45	**0**	8.22	7.06	0.72	0.04	0.56	0.002659

Supplementary Table 6. Comparing fitting models to the prokaryote abundance distribution for the mesocosm experiment in the single dose Nitrate-Phosphate-Silicate addition condition (C5). The delta Akaike Information Criterion is reported for the prokaryote diversity obtained for the indicated day. The delta Akaike Information Criterion (ΔAIC) indicates the most likely fit (value **0** in bold) and the difference to the most likely fit. For the six cases reported, the most likely fit is a distribution with a power law decay (either pure or truncated). The parameters of a power law distribution $P(x) \sim x^{-1-\alpha}$ are the scaling exponent α; for the truncated power law $P(x) \sim x^{-1-\beta} \exp(-\lambda x)$, are the scaling exponent β, and the characteristic abundance λ (λ=0, for a pure power law). ΔAIC PL: delta Akaike Information Criterion for power-law fit; ΔAIC TPL: delta Akaike Information Criterion for truncated power-law fit; ΔAIC LN: delta Akaike Information Criterion for log-normal fit; ΔAIC W: delta Akaike Information Criterion for Weibull fit. The standard error of the power law scaling exponent (α) is also reported.

	AIC PL	AIC TPL	AIC LN	AIC W	α	standard error (α)	β	λ
Day 1	*0*	1.21	4.27	8.33	0.57	0.04	0.54	0.000118
Day 2	*0*	0.83	2.07	2.26	1.34	0.26	1.37	0.00891
Day 3	4.2	**0**	11	8.85	0.54	0.03	0.43	0.000943
Day 4	**0**	0.74	6.24	12	0.57	0.03	0.56	0.000038
Day 5	19	**0**	39	36	0.45	0.02	0.43	0.000383
Day 6	**0**	0.84	4.44	4.34	0.60	0.04	0.56	0.000138
Day 7	**0**	0.15	7.61	8.27	0.57	0.03	0.52	0.000241
Day 8	11	**0**	23	22	0.46	0.03	0.42	0.000608
Day 9	14	**0**	28	26	0.41	0.02	0.32	0.00086
Day 10	**0**	1.89	3.04	3.09	1.00	0.15	0.86	0.012531
Day 11	6.45	**0**	2.23	2.34	1.15	0.33	1.59	0.144513
Day 12	**0**	0.97	6.36	6.64	0.75	0.06	0.73	0.000579
Day 13	**0**	1.83	2.42	5.32	0.79	0.06	0.77	0.000053
Day 14	**0**	1.29	4.92	4.6	0.68	0.05	0.63	0.000336
Day 15	**0**	1.91	2.13	2.61	0.98	0.16	0.91	0.000611
Day 16	0.41	**0**	7.98	7.53	0.62	0.04	0.51	0.00157
Day 17	9.15	**0**	18	15	0.53	0.04	0.37	0.010179
Day 18	6.95	**0**	9.97	8.52	0.61	0.03	0.45	0.001478
Day 19	10	**0**	16	17	0.54	0.04	0.49	0.003685
Day 20	1.08	**0**	10	9.44	0.65	0.04	0.51	0.002964

Supplementary Table 7. Comparing fitting models to the prokaryote abundance distribution for the mesocosm experiment in the single dose Nitrate-Phosphate-Silicate addition condition (C6). The delta Akaike Information Criterion is reported for the prokaryote diversity obtained for the indicated day. The delta Akaike Information Criterion (ΔAIC) indicates the most likely fit (value **0** in bold) and the difference to the most likely fit. For the six cases reported, the most likely fit is a distribution with a power law decay (either pure or truncated). The parameters of a power law distribution $P(x) \sim x^{-1-\alpha}$ are the scaling exponent α; for the truncated power law $P(x) \sim x^{-1-\beta} \exp(-\lambda x)$, are the scaling exponent β, and the characteristic abundance λ (λ=0, for a pure power law). ΔAIC PL: delta Akaike Information Criterion for power-law fit; ΔAIC TPL: delta Akaike Information Criterion for truncated power-law fit; ΔAIC LN: delta Akaike Information Criterion for log-normal fit; ΔAIC W: delta Akaike Information Criterion for Weibull fit. The standard error of the power law scaling exponent (α) is also reported.

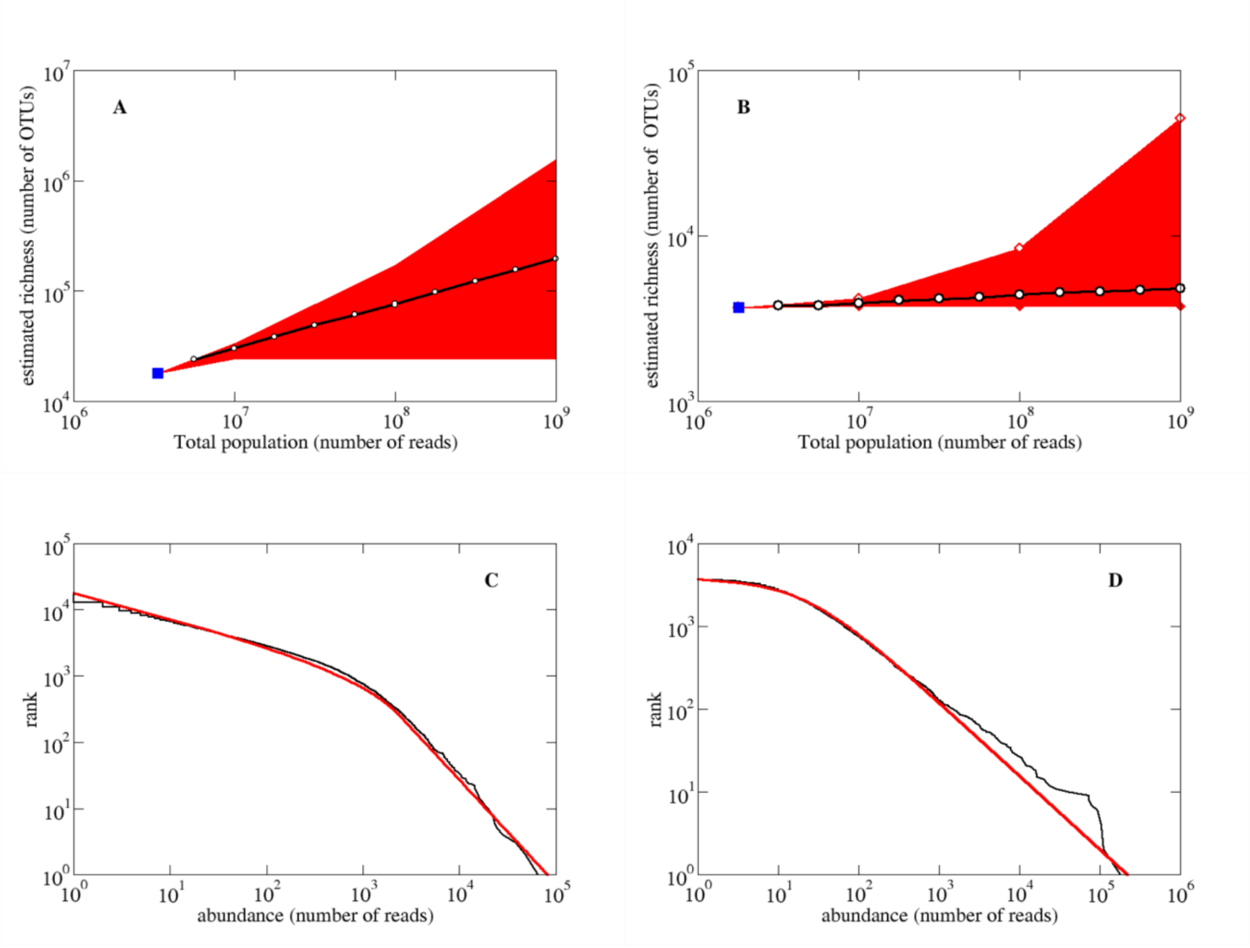

Supplementary Figure 1. Estimation of the prokaryote diversity for the (A) upper and (B) deep ocean. (**A**) In the upper ocean, the estimation of the prokaryote diversity is calculated (i) following the upper D_+ (open diamond) and lower D_- (filled diamond) estimator of Ref. (13); and (ii) with the abundance distribution obtained by fitting to the empirical data: double power-law function (filled circles). The distribution was extended to a greater number of reads as explained in the methods of the main text. (**C**) For the upper ocean the abundance distribution is modeled as $P(x) \sim x^{-1-\delta}$, for $x < x_c$; and $P(x) \sim x^{-1-\alpha}$, for $x \geq x_c$, with the parameter values changing as described in the methods of the main text. The black solid line shows the abundance-rank distribution for the aggregated data from the TARA Oceans Expedition, while the red line shows the fitted distribution. The scaling of the expected diversity depends on the transition point: when the transition point is fixed, increasing the total population enlarges the regime with the fastest decay (α) and the scaling of the prokaryote diversity grows accordingly ($S \sim N$); when the transition point increases linearly, the regime with the smallest decay (δ) and the scaling of the prokaryote diversity grows accordingly ($S \sim N^{\delta}$). (**B**) In the deep ocean, the estimation of the prokaryote diversity is calculated (i) following the upper D_+ (open diamonds) and lower D_- (filled diamonds) estimator of Ref. (13); and (ii) with the abundance distribution fitted to a shifted power law distribution (filled circles) $P(x) \sim (x+x_0)^{-1-\alpha}$, with exponent α=0.89 and the shift x_0=20.34. The empirical data are indicated as a blue square. (**D**) Abundance-rank distribution for the upper ocean used to expand the empirical distribution to larger populations in (**C**). The black solid line shows the abundance-rank distribution for the aggregated data from the Malaspina Expedition, while the red line shows the fitted distribution.

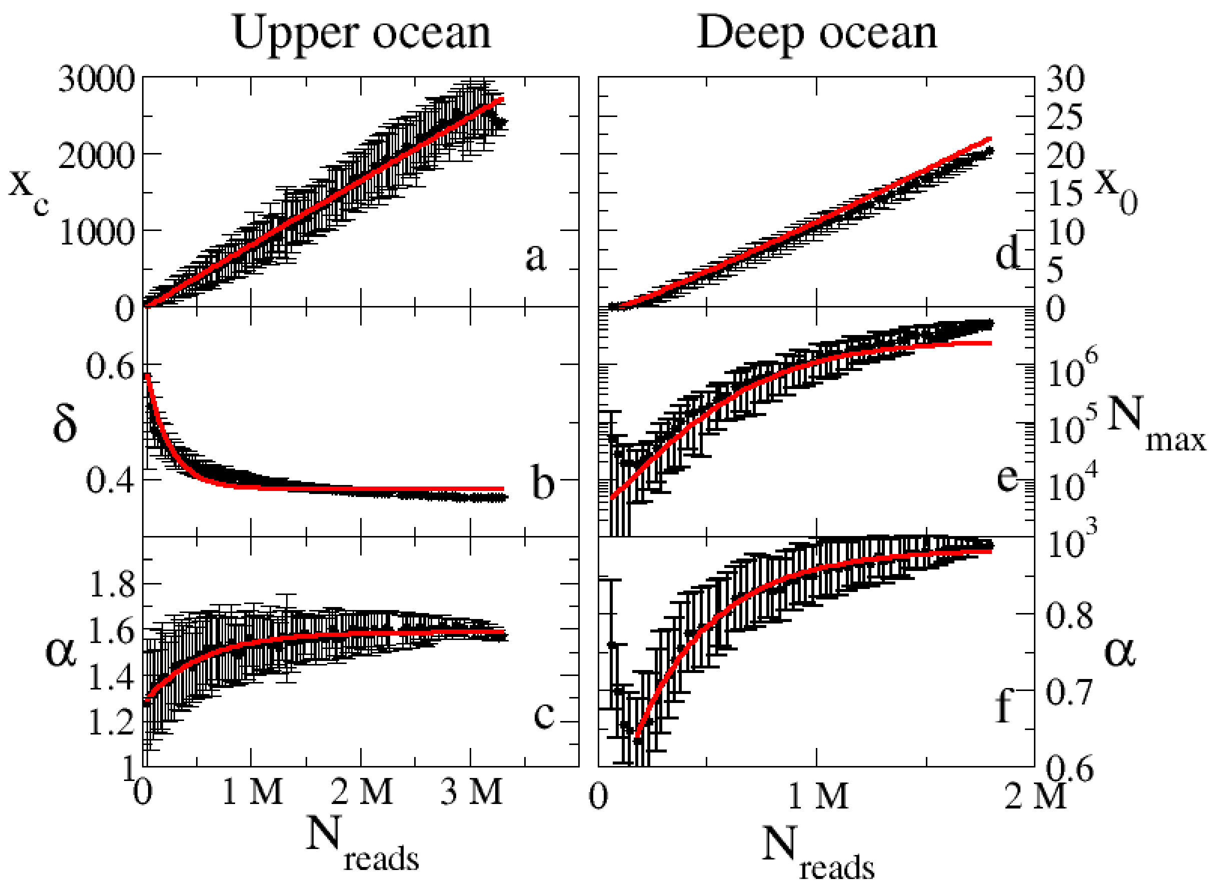

Supplementary Figure 2. **Fitting of parameters for the distribution of prokaryote diversity.** On the left column, we show the parameters fitting the double power law distribution for the upper ocean x_c **(a)**, δ **(b)** and α **(c)** as a function of the number of reads. On the right column we show the parameters of the shifted power law distribution fitted to the data from the deep ocean x_0 **(a)**, N_{max} (b) and α **(c)**. Black circles represent the average of the parameters for different resamplings of the data, while the red line shows the fit to the parameters that will let us extrapolate the parameters to larger values of the number of reads. The upper ocean parameters were found to behave approximately as $x_c = 0.0002 \cdot N_{reads}^{1.1} + 52.6$, $\delta = 0.32\ (1 + 0.71\ exp(- N_{reads} / 570007))$ and $\alpha = 1.42\ (1 - 0.2\ exp(- N_{reads} / 110185))$. For the deep ocean $x_0 = 0.000003\ N_{reads}^{1.1} - 1$, $\alpha = 0.88\ (1 - 0.45\ exp(- N_{reads} / 363263))$ and $<x> = 0.00042\ N_{reads}^{0.97} + 23.6$
